# Knockdown Indian Hedgehog (Ihh) does not delay Fibular Fracture Healing in genetic deleted Ihh mice and pharmaceutical inhibited Ihh Mice

**DOI:** 10.1038/s41598-018-28657-7

**Published:** 2018-07-09

**Authors:** Shengchun Li, Chuan Xiang, Xiaochun Wei, Hongbin Li, Kai Li, Xiaojuan Sun, Shaowei Wang, Min Zhang, Jin Deng, Xiaodu Wang, Pengcui Li, Ruifang Li, Yanxiang Zhang, Lei Wei

**Affiliations:** 1 The Second Hospital of Shanxi Medical University, Taiyuan, 030001 China; 2 Shaoxing People’s Hospital, Shaoxing, China; 3 Affiliated Hospital of Guizhou Medical University, Guiyang, China; 4 University of Texas at San Antonio, San Antonio, TX USA; 5 The third people’s Hospital of Hubei Province, Wuhan, China; 6 Department of Orthopedics, Warren Alpert Medical School of Brown University/RIH, Providence, RI USA

## Abstract

The objective of this study was to determine if Ihh is required for fracture healing. Fibular fracture was created in adult Col2a1-CreER^T2^; Ihh^fl^^/^^fl^ mice. Ihh^fl^^/^^fl^ mice received Tamoxifen (TM) to delete Ihh. WT mice received Cyclopamine to inhibit Hh pathway. Callus tissue properties and Ihh pathway were analyzed at 1, 2, and 3 weeks post-fracture by X-ray, micro-CT, mechanical test, RT-PCR and immunohistochemistry. Deleted Ihh was evidenced by the occurrence of growth plate closure in the Ihh^fl/fl^ mice by X-ray 3 weeks after TM treatment. All mice showed fracture healing at 3 weeks post-operation. Histology analysis indicated that, compared to the control, cartilage area was less in fracture sites from Ihh deficient animals by either genetic deletion or drug inhibition at 1 and 2 weeks post-fracture. Ihh immunostaining and its mRNA level were diminished in the fracture callus in Ihh reduced mice. There was no significant difference in BV/TV, BMD and mechanical test. Interruption to Ihh pathway by either genetic or pharmaceutical approach didn’t affect fibular fracture healing in these mice. This surprised finding implicates that the deleted Ihh does not affect fracture healing in this model.

## Introduction

The fracture healing is a complex and regenerative process, which closely recapitulated the steps involved in fetal endochondral ossification^1–4^. Recent studies have suggested that the genetic mechanisms regulating embryonic endochondral ossification also regulate postnatal bone growth^5,6^.

Collective evidences have demonstrated that Hedgehog signaling pathway plays a critical role in developmental processes and postnatal homeostasis of many tissues, including the bone and cartilage^7–10^. Hedgehog family protein in mammals includes Sonic hedgehog (Shh), Indian hedgehog (Ihh), and Desert hedgehog (Dhh). The Hh proteins share a common signaling pathway. They bind to the receptor Patched (Ptch), which prevents the inhibition of the signal transduction protein Smoothened (Smo), activating downstream transduction pathway. In the absence of hedgehog protein, Ptch inhibits the activity of Smo. The downstream components of the signaling pathway are the glioblastoma-associated protein (Gli) family of transcription factors: Gli1, Gli2, and Gli3^11^. The role of the Hedgehog (Hh) pathway to postnatal skeletal homeostasis is still under controversy. Recent papers by Ohba *et al*.^12^ and Mak *et al*.^13^ showed opposite effects. With the objective of addressing the role of the enhanced Hh signaling in postnatal bone cells, both Ohba and Mak’s studies used genetic mouse models to delete Patched (Ptch), the membrane-bound molecule that is downstream of Hh and acts as a functional inhibitor of Hh signaling. Ohba *et al*. demonstrated that enhanced Hh signaling increased bone mass, while Mak *et al*. showed that enhanced Hh signaling led to decreased bone mass. The apparent discrepancies between the two papers beg for further studies.

Ihh null mutant mice displayed markedly reduced chondrocyte proliferation, maturation of chondrocytes at inappropriate position, and a failure of osteoblast development in endogenous bone formation and growth development^1,14^. Ihh secreted by the prehypertrophic chondrocytes controls the onset of osteoblast differentiation in the endochondral skeleton^4^. Murakami and his colleagues findings indicate that Ihh is expressed in prehypertrophic chondrocytes and also by early hypertrophic chondrocytes during fracture healing in adult animals, and suggest that it may participate in the regulation of the endochondral bone formation of the fracture site^15^. Tatsumi H and his colleague also found the number of Ihh-positive cells in the fracture area increased significantly up to 2 weeks after fracture, and then gradually decreased until 8 weeks after bilateral mandibular condylar fractures in a rat model^16^. These data suggested Ihh protein may play a critical role during fracture repair in adult animals in addition to its role in endochondral bone formation of growth plate development and postnatal bone formation. The Hh family of proteins plays fundamental roles in development and is conserved from animal to humans^7,8,17^. Given Ihh is critical to endochondral bone formation and the express of Ihh is up-regulated at bone fracture site, we hypothesize that Ihh may play a critical role in fracture healing. To test our hypothesis, we specifically delete Ihh to test its function using Col2a1-CreER^T2^; Ihh^fl^^/^^fl^ mice and Ihh inhibitor.

Aberrant activation of the hedgehog signaling pathway which is normally quiescent in the adults plays an important role in the pathogenesis of various types of cancers including skin, mammary gland, brain, lung and prostate^18^. The hedgehog inhibitors are a promising group of drugs to be used in human cancer chemotherapy. Cyclopamine, the most clinically advanced hedgehog pathway inhibitor has been tested for humans^19^. We assessed mice fibular fracture healing after administration of cyclopamine. We used Cyclopamine to confirm the genetic Ihh knockout result. The data from the study will also suggest whether hedgehog inhibitor could be continued to use in the carcinoma patients under fracture condition.

Surprisingly, we found that the deletion of Ihh and inhibition of Hh signaling pathway by its inhibitor have no effects on the fibular fracture healing. The finding indicates the level of Ihh may not be a critical factor during fracture healing in this model. Thus, the carcinoma patients may continue to treat with Hh inhibitor therapy if the patients have a fracture in future.

## Materials and Methods

### Animals

*Col2a1-CreER*^*T2*^; Ihh^fl/fl^ mice were supplied by Beate Lanske, and bred as previously described^10^. Two-month-old *Col2a1-CreER*^*T2*^; Ihh^fl/fl^ mice were randomized into the experimental group (n = 45) (deleted Ihh by injecting Tamoxifen (TM): 1 mg/10 g/day for 5 consecutive days) and the control group (n = 45) (injected corn oil). To keep the activity of the TM-inducible Ihh deletion, additional TM (1 mg) was given every week until the end point. To validate the genetic loss-of-function results, 10-week-old C57/B6 WT mice (N = 24) were randomized into Ihh inhibition group by Cyclopamine (25 mg/kg body weight/day for 3 weeks) and control group by hydroxypropyl-b-cyclodextrin for 3 weeks. (This study was approved by Rhode Island Animal Welfare Committee, 0112-09. All experiments were performed in accordance with the Public Health Service (PHS) Policy and the Guide to the Care and Use of Laboratory Animals).

### Surgical Procedure

The mice were anesthetized by intraperitoneal injection (6 μl/g body weight) of 10 mg/ml ketamine and 0.25 mg/ml medetodamine, which provided approximately 30 minutes of deep anesthesia. The right legs were shaved and scrubbed with a 10% povidone-iodine solution to prepare for surgery. A 5-mm longitudinal skin incision was made on the right hind limb of all mice. Muscles were separated bluntly, exposing the lateral aspect of the fibula. A fracture was created with dissection scissors about 5 mm from fibular head and no internal fixation was used. Skin was closed with 6–0 Ethilon suture.

### Radiographs

The X-rays were taken to monitor fracture healing and growth plate closure every week for 6 weeks for the Col2a1-cre ER^T2^ Ihh^fl/fl^ mice under anesthetized condition. The X-rays were taken at 0, 3 and 6 weeks for both cyclopamine-treated and control mice.

### Micro-CT scan

The muscle tissue of the fibulas was removed immediately after the mice were euthanized. The fibulas were fixed in 10% neutral buffered formalin for 24 h, followed by washing with phosphate-buffered saline and stored in 70% ethanol until scanning. The structure and mineralization of the fibular fracture callus were quantified by micro-computed tomography (μCT) at 2 weeks and 3 weeks after fracture (N = 24).

All scans were done using μCT 40 scanner under high resolution of tube settings of 55 kilovolt peak of energy, 145 microamperes of current with an integration time of 150 milliseconds in a tube of diameter 8 mm. Images of individual scan slices or reconstructed bones were captured as TIFF images. On each 2D tomogram located between the proximal and distal boundaries of the callus, semi-automated image segmentation was used to define the outer boundary of the callus and the periosteal surface of the cortex. The volume of interest was the region enclosed by these boundaries and the 3-D structure was reconstructed.

According to the semi-automated image, histomorphometrically measurements of the following measures of callus structure and composition were evaluated from the μCT image data for each specimen: total callus volume (TV), mineralized callus volume (BV), callus mineralized volume fraction (BV/TV) and bone mineral density (BMD expressed as milligrams of hydroxyapatite (HA) per cubic cm) were calculated by using the slices of the scanned callus, and by using the manufacturer’s 3D analysis software.

### Histology and immunohistochemistry

The fibular fracture callus were carefully dissected for a histological analysis at 1 week and 2 weeks post-fracture (N = 12) after the mice were sacrificed. Each fibula was fixed in 4% paraformaldehyde at 25 °C for 48 hours, decalcified in 20% ethylene diamine tetraacetic acid (EDTA) at 37 °C for 14 days, then embedded in paraffin. The embedded samples were cut longitudinally. 5μm thick sections were collected and stained with Safranin O/Fast Green.

Immunohistochemistry was used to detect the expression of Ihh from week 1 and week 2 callus sections. The slides were deparaffinized, rehydrated and rinsed with double-distilled water. Slides were blocked in 3% hydrogen peroxide (Sigma-Aldrich, St Louis, MO) in methanol (Sigma-Aldrich, St Louis, MO) for 30 mins. They were treated with 5 mg/ml hyaluronidase in PBS (Sigma-Aldrich, St Louis, MO) for 20 mins, rinsed three times with double-distilled water. Slides were incubated overnight at 4 °C with a polyclonal antibody against Ihh at 1:500 dilution (sc-1196, Santa Cruz, Santa Cruz, CA). The negative control sections were incubated with isotype control (sc-1196-P, Santa Cruz, Santa Cruz, CA) in PBS. The specificity of Ihh antibodies used in this study has been validated by immunofluorescence stain and Western blot^20^. DAB immunohistochemistry kit (Invitrogen Corporation, 542 Flynn Road, Camarillo, CA,93012 USA) was used to stain according to the protocol supplied by Invitrogen.

### Mechanical test

At 3 weeks post-fracture, 6 mice from each group were euthanized and the entire fibulas were dissected free from soft tissues and preserved in gauze with saline and stored at −20 °C until testing. The measurement of the cross-section of the middle callus for each sample by nanoindentation modulus was performed by the University of Texas Health Science Center at San Antonio^21–23^. Two nanoindentation tests for each bone sample were performed on the posterior side of the left femur at room temperature and humidity using an MTS Nano Indenter system (Nano Indenter XP, MTS Systems Co., Oak Ridge, TN) in a load-control mode. A Berkovich indenter was used to penetrate the specimen surface with a maximum penetration depth of 2 μm and a rate of 25 μN/s. The indenter was loaded and unloaded, with one intermediate constant-load holding period at the peak load before unloading to alleviate the effect of viscoelastic deformation. Elastic modulus and hardness was calculated from the unloading curve using the Oliver–Pharr method^24,25^. The elastic modulus gives us an idea on how rigid the tissue is, and the hardness measures the resistance of the tissue to the indentation deformation. The former gives an estimate of local mineralization and the latter is the manifestation of the tissue quality.

### Real-time PCR

Fibular fracture calluses were harvested from 6 mice in each group after 7 and 14 days post fracture. Total RNA was extracted from each sample used RNeasy Mini Kit (QIAGEN Sciences, Maryland 20874, USA), cDNA was synthesized with the iScript^TM^ cDNA synthesis Kit (Bio-Rad Laboratories, Inc. 2000 Alfred Nobel Drive, Hercules, CA 94547). The mRNA levels of Ihh, Gli1, Runx2, BMP2, VEGF, osteocalcin, collagen I and collagen II were quantified by Real-time polymerase chain reaction (RT-PCR) using the iCycler RT-PCR Detection System (Bio-Rad, Hercules, CA, USA). PCR reactions were carried out in a final volume of 25 μl, with 1 μl of cDNA sample, 1 μl of each primer, and Quantitect® SYBR® Green master mix (TaKaRa BIO). The PCR conditions were 40 cycles at 95 °C denaturation for 10 s, 55 °C annealing for 30 s, and 72 °C extension for 30 s. The endogenous control 18S rRNA was previously determined to be the best housekeeping gene for mechanobiology of cartilaginous tissues^26^. The transcript level of Gli 1 mRNA was used to show whether Ihh had been knockdown. The other genes were used to explain the results of the X-ray, micro-CT and mechanical test. Specific primers designed from the published sequence were listed in Table 1. All genes from each sample were run in triplicates and analyzed by the 2^−(ΔΔCt)^ method.

**Table 1:** Primer sequences.

Gene	Forward Primer	Reverse Primer
Gli 1	5′-CTTCAAGGCCCAATACATGC-3′	5′-ATGGCTTCTCATTGGAGTGG-3′^40^
Runx2	5′-CATGGTGGAGATCATCGC-3′	5′-ACT CTT GCC TCG TCC ACT C-3′^41^
Col-1	5′-TCCGACCTCTCTCCTCTGAA-3′	5′-GAGTGG GGTTATGGAGGGAT-3′^41^
Osteocalcin	5′-GAACAGACTCCGGCGCTA-3′	5′-AGGGAGGATCAAGTCCCG-3′^42^
VEGF	5′-ATGGACGTCTACCAGCGAA-3′	5′-ACTGTTCTGTCAACGGTGA-3′^42^
18srRNA	5′-CGGGTCATAAGCTTCGTT-3′	5′-CCGCAGGTTCACCTACGG-3′^42^
Ihh	5′-GGCTTCGACTGGGTGTATTA-3′	5′-CGGTCCAGGAAAATAAGCAC-3′^40^
BMP2	5′- AGCGTCAAGCCAAACACAAACAG-3’	5′-GGTTAGTGGAGTTCAGGTGGTCAG-3’^43^
Col-2	5′-AAG GGACAC CGA GGT TTC ACT GG-3′	5′-GGG CCT GTT TCT CCTGAG CGT-3′^44^

### Statistics analysis

Statistical analysis was performed by the Student’s t-test in a minimum of 6 individuals for micro-CT, mechanical test and real time PCR. A p-value < 0.05 was considered statistically significant.

## Results

### Deletion of Ihh was confirmed by IHC, RT-PCR and X-ray in mice fibular fracture model

**Figure 1:**
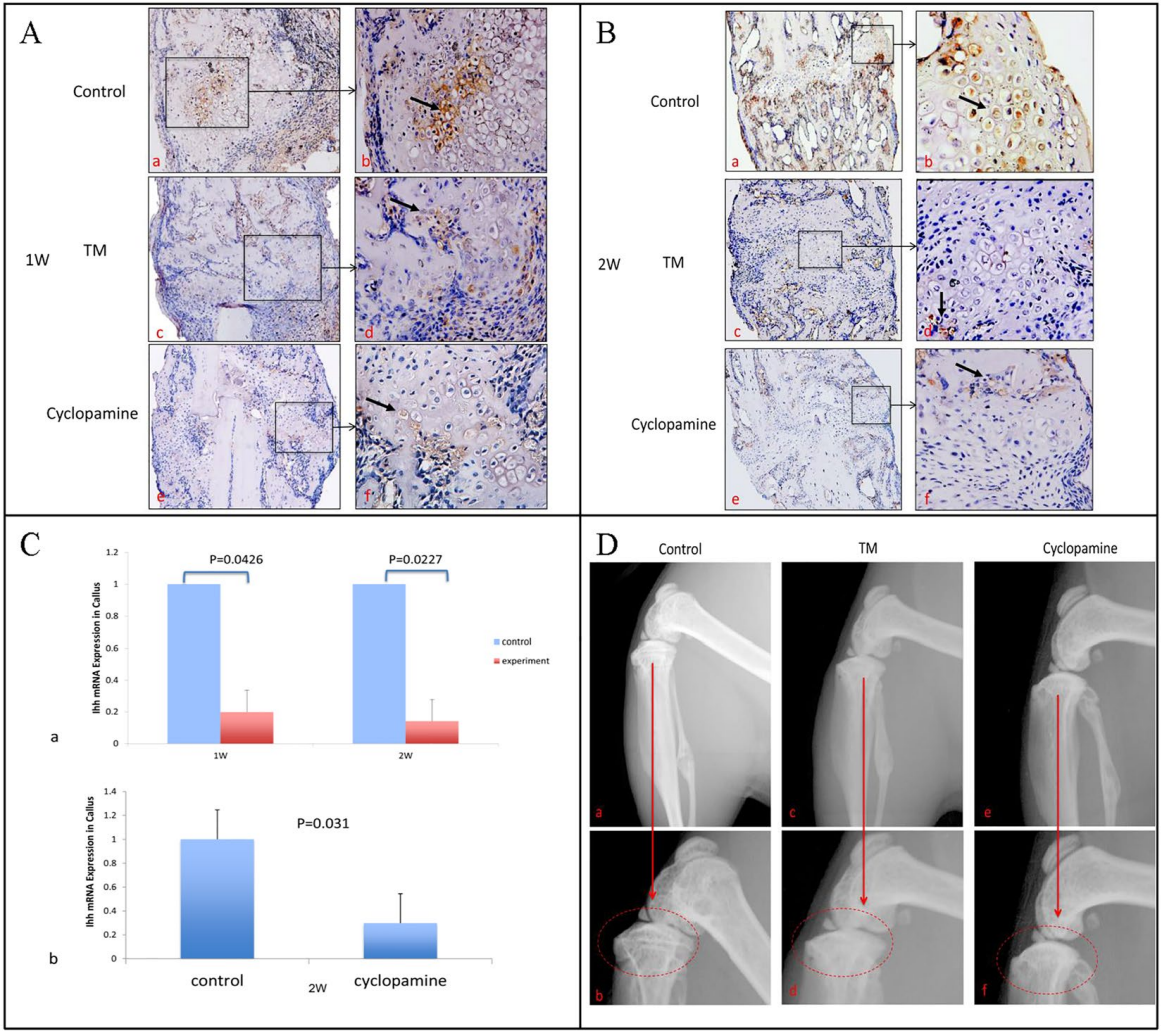
Deletion and inhibition of Ihh signaling was demonstrated by IHC, RT-PCR analysis and X-ray. The fibulas of 2-month-old Ihh deletion mice (*Col2a1-CreER*^*T2*^; Ihh^fl^^/^^fl^), Cyclopamine treatment in wild type (WT) C57/B6 mice, and controls were fractured and the expression of Ihh from the calluses harvested at 1-week (**A**), and 2 weeks (**B**) post fracture was detected by IHC staining. Strong staining of Ihh was observed in the control group compared with TM and Cyclopamine treated group. (**C**) RNA from the fracture calluses was analyzed for transcript levels of the Ihh mRNA. Ihh mRNA was decreased 5-fold at 1-week and 6-fold at 2-weeks post fracture from the calluses in TM group and 3-fold at 2-weeks post fracture from the calluses in Cyclopamine treated group when compared to the normal control samples. (**D**) Growth plate was investigated by radiographic analysis in Ihh deletion mice at 3 weeks post administration of TM, Cyclopamine and controls. Closure of growth plate has been observed at 3-weeks post fracture in TM and Cyclopamine treated group.

To confirm the efficiency of Ihh deletion in calluses, we harvested fracture calluses at one- and two-weeks post fracture for immunohistochemistry (IHC) staining of Ihh. Additionally, at the one and two-weeks post fracture, total RNA was isolated from the fracture calluses and RT-PCR was used to quantify transcript levels of the Ihh mRNA. Ihh expression determined by IHC staining in fracture calluses was decreased in TM group compared with the control group (Fig. 1A,B). Ihh mRNA was decreased 5-fold at 1-week and 6-fold at 2-weeks post fracture from the calluses in TM group when compared to normal control samples (Fig. 1C). These data confirm Ihh gene was successfully deleted by administration of TM in the *Col2a1-CreER*^*T2*^; Ihh^fl^^/^^fl^ mice. Another direct evidence of Ihh deletion is closure of growth plate^27,28^, which was observed at 3-weeks post fracture in TM group determined by X-ray (Fig. 1D).

### Fibular fracture was healed in both the genetic deletion of Ihh and Hh inhibitor treated animals

**Figure 2:**
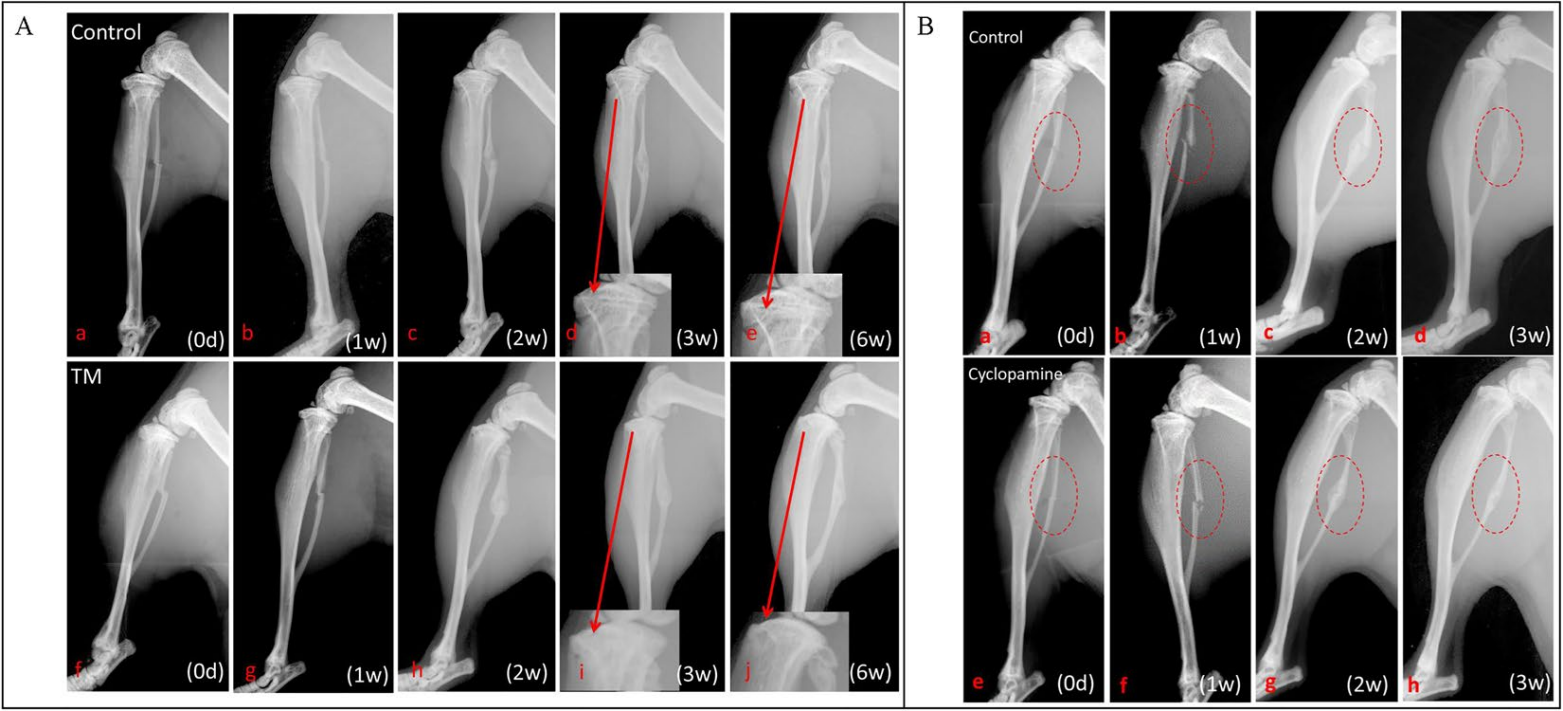
Deletion and inhibition of hedgehog signaling in chondrocytes does not affect fracture repair by X-ray. (**A**) Fibular fracture healing was investigated by radiographic analysis in TM and control group at 0-, 1-, 2-, 3-, and 6-week post fracture. (**B**) Fibular fracture healing was investigated by radiographic analysis in Cyclopamine treatment and control groups at 0-, 1-, 2- and 3-week post fracture. There is no significant difference for fibular fracture healing among the three groups.

**Figure 3:**
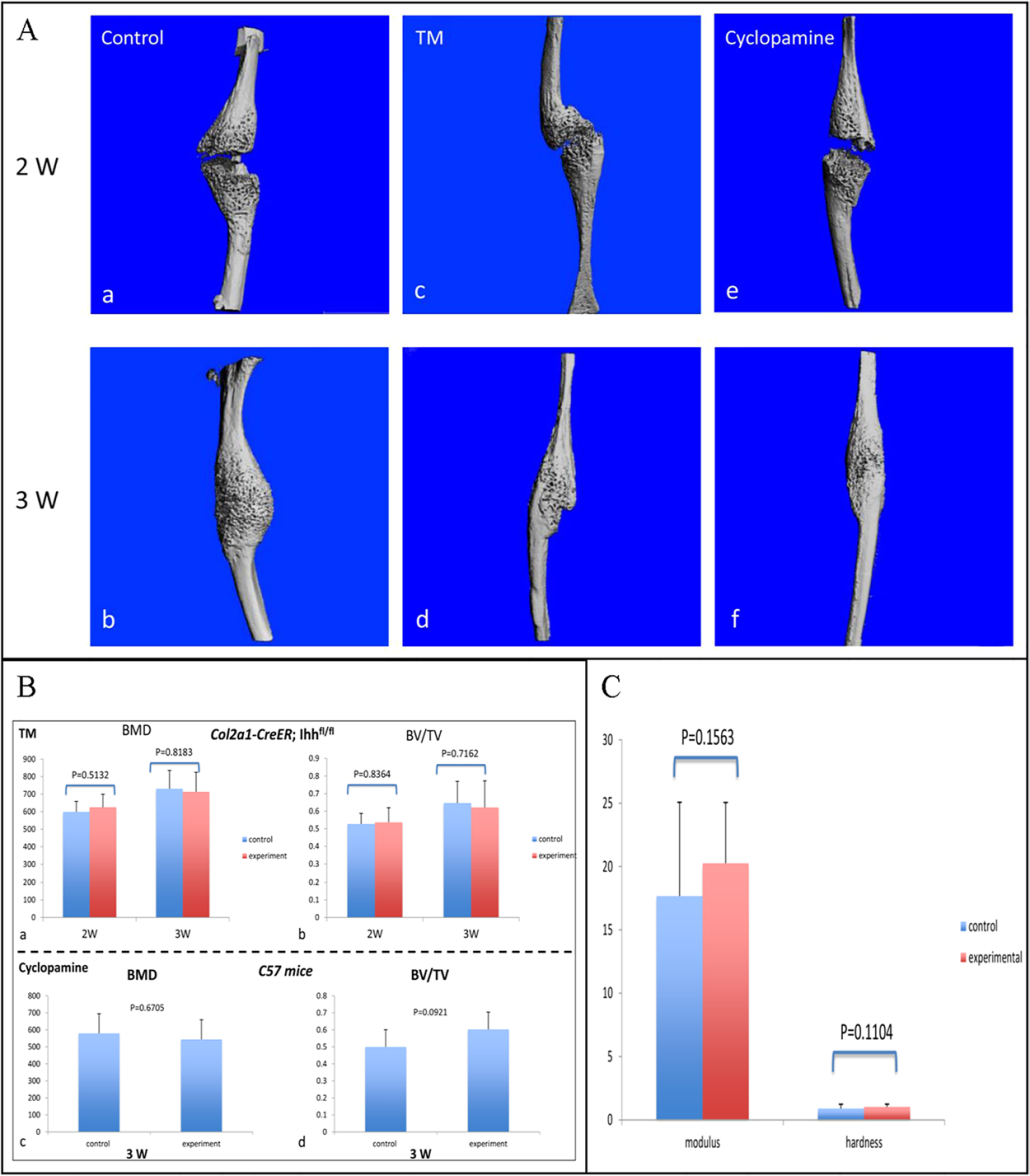
Deletion and inhibition of hedgehog signaling in chondrocytes does not affect fracture repair detected by micro-CT and mechanical test. (**A**) Representative micro-CT scanning at fracture site after 2 and 3 weeks of fracture healing in a control, a TM-treated and a cyclopamine treated animals. Bone tissue was quantified using micro-CT (**B**) as percentage of bone mineral density (BMD) and bone volume deposited relative to total callus volume (BV/TV). There was no significant difference either on BMD or BV/TV among the three groups. (**C**) Mechanical properties of bone calluses in control and TM-treated mice at 3 weeks post-fracture were assessed by elastic modulus and hardness using nanoindentation tests. There are similar nanoindentation test results between the two groups. Data is shown as means and error bars represent 95/% confidence intervals.

The fibular fracture was healed at week three post-fracture determined by X-ray and micro-CT in both the genetic deletion of Ihh mice and Hh inhibitor treated mice. Radiographic analysis of *Col2a1-CreER*^*T2*^; Ihh^fl^^/^^fl^ mice indicated fracture healing was similar with that of controls (Fig. 2A) at 1-, 2-, 3- and 6-weeks post fracture. Micro-computed tomography (Micro-CT) analysis and histomorphometry data showed a similar pattern between the *Col2a1- CreER*^*T2*^; Ihh^fl^^/^^fl^ fracture calluses and the control mice (Fig. 3A,B). Mechanical test data of *Col2a1-CreER*^*T2*^; Ihh^fl^^/^^fl^ mice revealed the intensity of fracture healing was also similar with that of controls (Fig. 3C). These data indicated that the deleted Ihh signaling did not affect the fibular fracture repair.

### Deletion of Ihh signaling had a less endogenous bone formation during fibular fracture healing

**Figure 4:**
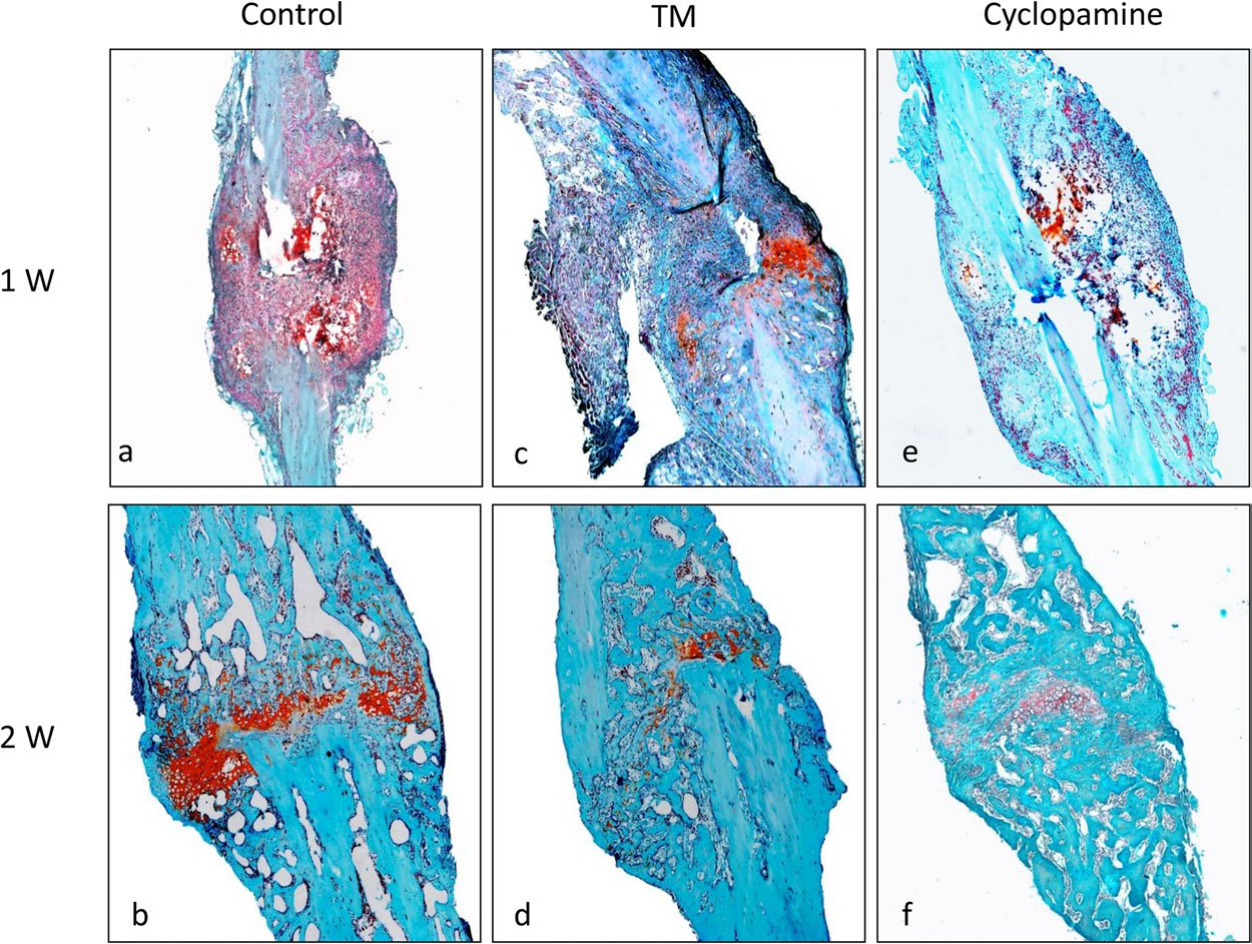
Deletion and inhibition of Ihh signaling in chondrocytes lead to less endochondral bone formation determined by Safranin O staining. The amount of endochondral bone formation in control, TM and cyclopamine treated animals at 1-, and 2-week post fracture was analyzed by histology (Safranin-O/Fast Green). A strong Safranin O staining was observed in the control group compared with TM and Cyclopamine treated groups.

The intensity of safranin O staining is directly proportional to the proteoglycan content during chondrogenesis in normal cartilage formation^29^. The endogenous bone formation is main component during fracture healing. To observe endogenous bone formation after Ihh deletion, *Col2a1-CreER*^*T2*^ was used to drive expression of the conditional alleles in a chondrocyte-specific manner during fracture repair *in vivo*. Histological analysis confirmed the amount of chondrogenesis in Col2a1-*CreER*^*T2*^; Ihh^fl^^/^^fl^ mice fracture calluses was less than that of controls after 1- and 2-weeks post fracture (Fig. 4).

### Deletion of Ihh from the callous decreased the level of Gli1 and Collagen II but did not affect the mRNA level of BMP-2, VEGF, Collagen I and osteocalcin

**Figure 5:**
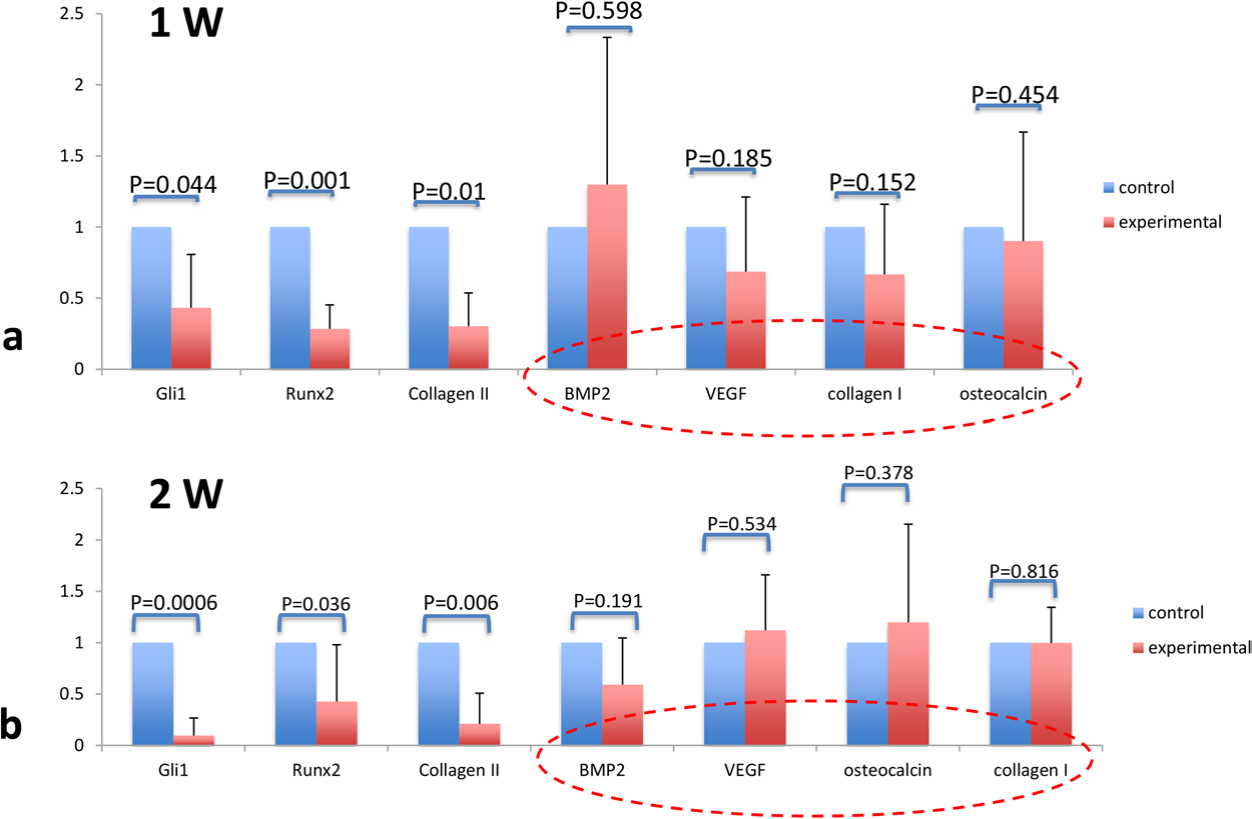
Inhibition of Ihh signaling in chondrocytes decreased chondrocyte-related gene expression but did not affect osteoblast-related gene expression. RNA from the calluses at 1- (**a**) and 2-week (**b**) post fracture was analyzed for transcript levels of Gli1, Runx2, Collagen II, BMP-2, VEGF, Collagen I and Osteocalcin from the control and the deleted Ihh mice. Levels of Gli1, Runx2 and Collagen II mRNA were high in the control group compared with TM group. While the mRNA levels of BMP-2, VEGF, Collagen I, and Osteocalcin showed no significant difference between the control and TM group. Bar graph showing a relative fold change of mRNA expression using means and error bars represent 95% confidence intervals.

Total RNA was isolated from the fracture calluses at 7- and 14-days post fracture and RT-PCR was used to quantify transcript levels of Gli1, Runx2, Collagen II, BMP-2, VEGF, Collagen I, and osteocalcin. Interestingly, RT-PCR data from the early-stage of fracture repair, 1- and 2-weeks following injury, a time when chondrogenesis is most active, showed deletion of Ihh signaling in chondrocytes leads to decreased Gli1, Runx2 and Collagen II expression and there was no significantly change related to BMP-2, VEGF, Collagen I, and osteocalcin compared to that of controls (Fig. 5). The decrease of the hedgehog pathway targeted genes Gli 1 in experimental group also directly verified that the deletion of Ihh in our animal model was successful.

### Pharmaceutical Inhibition of Hedgehog Signaling has similar result as genetic deletion of Ihh

To verify our genetic deletion of Ihh, we induced fibular fractures in wild type (WT) C57/B6 mice treated by hedgehog pharmaceutical inhibitor Cyclopamine. The fracture calluses were harvested at 1- and 2-weeks post fracture for immunohistochemistry (IHC) staining of Ihh (Fig. 1A,B), transcript levels of the Ihh mRNA (Fig. 1C) and Safranin O (Fig. 4). We noticed there was a similar pattern as genetic deletion of Ihh. Radiographs of fibular fracture from mice treated by cyclopamine showed the fibular fracture healing was also similar with that of controls (Fig. 2B). There was a similar amount of calluses in the experimental mice treated by Cyclopamine compared with the controls determined by micro-CT (Fig. 3A,B).

## Discussion

To study the significance of chondrocyte-derived Ihh in fracture healing, we have selectively ablated the Ihh gene from collagen type II-expressing cells. Similar with previous studies^11,14,15^, closure of growth plate was observed in TM group by x-ray at 3 weeks post fracture, but was still clear in control group, which is indirectly evidence for successfully deleted Ihh in our models.

Unexpectedly, the deletion of Ihh in chondrocyte-specific manner did not affect fracture healing process in our study. This data demonstrates although Ihh signaling pathway in the chondrocytes plays a critical role in embryogenesis and growth plate development, the interrupt Ihh pathway does not delay fibular fracture repair in the adult mice. This result is in agreement with previous study in which Baht *et al*. reported that the deletion of *Smo* pathway in chondrocytes, a transducer of Ihh signaling pathway, did not alter the fracture repair phenotype^30^.

No external and internal fixation was performed for this study. Thus, the type of fracture healing for this model is endochondral bone formation which typically occurs to non-rigid fracture fixation^31,32^. Although previous studies have demonstrated the deletion of Ihh signaling in the chondrocytes leads to abnormal chondral bone and absence of osteoblastic differentiation^1,3–6,14,15^, our results demonstrated that the deletion of Ihh in chondrocytes did not alter fibular fracture healing process. Previous studies have demonstrated the chondrocyte is the principle source of Ihh, affecting both chondrocyte and osteoblast differentiation during endochondral skeletal development^33^. Additionally, Ihh secreted by postnatal chondrocytes is essential for bone growth, and Ihh produced by osteoblasts is not sufficient to compensate for this loss^14^. Ihh has been shown to be restrictedly expressed in chondrocytes and some of these osteoblasts near the endochondral ossification front during fracture healing^6,15^.

Thus, one possible explanation for these data is that Ihh signaling pathway in the chondrocytes may be acted as a “switching” molecular in fracture repair, and independent on the amount of Ihh expression. Ihh as “switch” molecular in the fracture healing process will explain why there is no delayed fracture healing even there is an obvious decrease of Ihh express detected by IHC and RT-PCR from the callous tissue.

We noticed that the amount of endochondral cartilage tissue decreased at 1- and 2-weeks post fracture determined by Safranin O staining from Ihh deleted mice compared with the control mice (Fig. 4). This is consistent with previous report in which the conditional deletion of Ihh from chondrocytes resulted in decreased endochondral bone formation^11^. Since the signals and mechanisms regulating fracture repair recapitulate the embryonic bone formation and postnatal growth^3,5^. In the present study, our PCR data including BMP-2, VEGF, Collagen I, and Osteocalcin didn’t show a significant difference between the deleted Ihh mice and control mice. Thus, another possible explanation for this data is that the decrease of endochondral bone formation may be compensated by intramembranous bone healing during the fibular fracture repair. Because the fracture healing is a complex interplay between the endochondral bone formation and the intramembranous ossification in which bone forms directly from the cortical bone and periosteum to bridge the fracture gap^23^.

Our RT-PCR analysis showed that the level of collagen II and aggrecan mRNA was decreased, but osteocalcin and collagen I mRNA were not affected in the early healing stages when compared to controls. These data further indicate that the deleted Ihh affects the chondrogenesis but does not affect the fracture healing process.

Additionally, Hh signaling pathway plays a key role in the carcinogenesis, including primary colon cancers, metastatic disease, human carcinoma xenografts^9^, and human colon carcinoma cell lines^34,35^. The inhibitor of hedgehog signaling pathway has been used to treat the carcinoma^36^. In addition, several Hh pathway inhibitors that target Smo have demonstrated single-agent efficacy in patients with ligand-independent tumors^6,13,15,16,37–39^. Cyclopamine was the first Smo inhibitor to be tested in humans. In this study, we demonstrated that the administration of cyclopamine did not affect fibular fracture healing process in the fracture model.

A potential limitation of this study is that the fibula is the slenderest bone of all the long bones in the vertebrate animals, which is not like the tibia that is a weight bearing bone. Another limitation of our study is that the mice are small animals. The data observed from the small animals may be not the same as the large animals. Thus, it is necessary to verify the finding using large animals before a clinical trial.

In summary, the data presented in this study indicates Ihh signaling may be a “switch” molecular during fracture healing and the deleted Ihh does not affect the fracture healing in this fibula fracture model.
